# Chronic purulent pericarditis: case report

**DOI:** 10.11604/pamj.2022.42.145.34018

**Published:** 2022-06-22

**Authors:** Proches Vara, Diana Urassa, Boniface Temba, Kajiru Kilonzo, Alex Mremi, Adnan Sadiq, Furaha Lyamuya

**Affiliations:** 1 Internal Medicine Department, Kilimanjaro Christian Medical Centre, Moshi, Tanzania,; 2 Pathology Department, Kilimanjaro Christian Medical Centre, Moshi, Tanzania,; 3 Radiology Department, Kilimanjaro Christian Medical Centre, Moshi, Tanzania

**Keywords:** Chronic pericarditis, purulent pericarditis, TB pericarditis, case report

## Abstract

Purulent pericarditis is an infection of the pericardial space that produces pus that is found on gross examination of the pericardial sac or on the tissue microscopy. In this case report, we will discuss a 31-year-old male who presented with a chief complaint of low-grade fevers, dry cough and difficulty breathing for about two weeks which preceded after removing of dental also two weeks prior. He was admitted and treated as COVID-19 in the isolation ward, he later developed cardiac tamponade and during pericardiocentesis thick pus was discharged. Pus culture and Gene Xpert tests were all negative. After his condition improved, the patient was transferred to the general ward with the pericardial window still discharging pus. Pericardiectomy was chosen as definitive management. The key takeaway in this report is that Empirical treatment with RHZE (rifampin, isoniazid, pyrazinamide, and ethambutol) in resource-limited settings is recommended due to difficulty in identifying the exact cause at a required moment.

## Introduction

Purulent pericarditis is an infection of the pericardial space that produces pus in the pericardial sac or on the tissue microscopy [1], its termed chronic when the disease is long-lasting (>6 months) and normally presents with pericardial fibrosis, thickening and scarring of the pericardium. In this case report, we will describe a 31-year-old patient with purulent pericarditis which was preceded with incision and drainage of dental abscess 2 weeks prior.

## Patient and observation

**Patient information:** a 31-year-old male (HIV negative), not known with any comorbidities, presented with 2 weeks history of difficulty in breathing, dry cough and low-grade fevers with progressive lower extremity swelling, exertional dyspnoea and air hunger at night. The symptoms started 2 weeks later after incision and drainage of a dental abscess. No history of chest trauma, B symptoms, no anaemia symptoms, no loss of consciousness, no change in micturition pattern. Other symptoms were unremarkable. He is a regular alcohol drinker and a non-smoker.

**Clinical findings:** on examination he was conscious, dyspnoeic, bilateral pitting oedema grade 4, saturation 97% 10L of O_2_, other vitals were stable. He had bilaterally reduced air entry, with distended neck veins, raised JVP by 6 cm, normal heart sounds. The abdomen was distended with positive shifting dullness.

**Figure 1:**
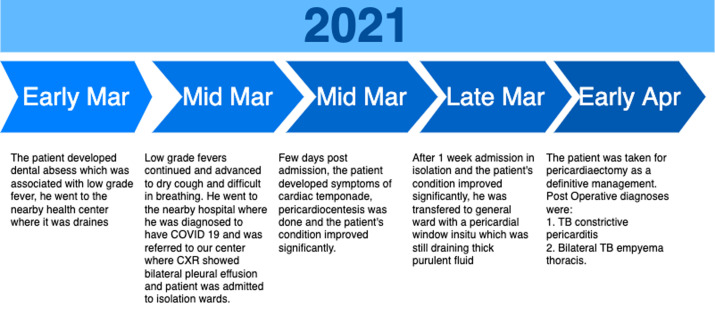
timeline of the patient's illness

**Timeline of current episode:** this can be seen in Figure 1.

**Figure 2:**
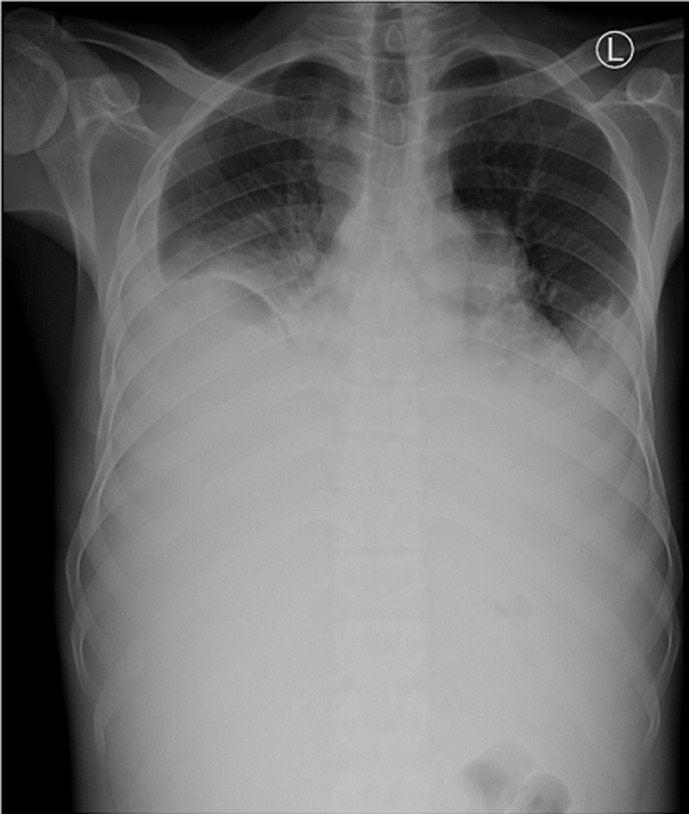
chest X-ray on admission showing massive bilateral pleural effusion

**Figure 3:**
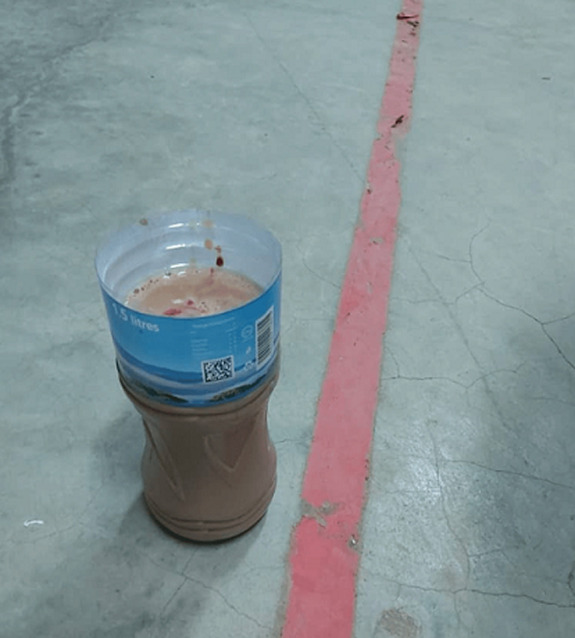
a bottle filled with pus collected after pericardiocentesis

**Figure 4:**
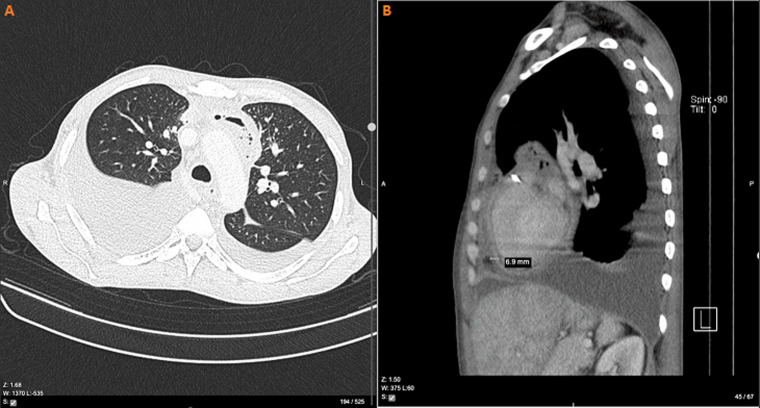
(A, B) CT scan of the chest showing bilateral partial atelectasis, more on the right lung and pericardial thickening of about 6.9 mm

**Diagnostic assessment:** blood workouts showed normocytic normochromic anaemia of 11.7, low albumin and protein with mild hyponatremia. Electrocardiogram (ECG) showed: sinus tachycardia with right axis deviation, chest X-ray (CXR) showed massive bilateral pleural effusion (Figure 2). The pleural tap showed ambercoloured 1.5L on the right and 400 MLS on the left. He was treated as a suspect of viral pneumonia with congestive heart failure with improvement (Lasix, Ceftriaxone Sulbactam, Ivermectin, Ascorbic Acid, Cholecalciferol, Dexamethasone, Zinc Sulphate and Colchicine). A few days after admission, the patient presented with signs of cardiac tamponade. An echocardiogram confirmed pericardial effusion. Pericardiocentesis was done, 950 mls pus drained (Figure 3), drainage was left *in situ* with improvement. AntiTB, prednisolone and omeprazole initiated. Culture and sensitivity, gene Xpert and ZN staining of pus were done which all came negative and there was no organism seen. Pericardial fluid glucose level was 1.01 mmol/L. CT Scan of the chest showed bilateral pleural effusion with partial atelectasis of both lungs right: 4.7L, left: 1.5L with thick pericardium about 6.9 mm (Figure 4), the pericardial tube was *in situ*, but no evidence of pericardial effusion.

**Diagnosis:** tuberculosis pericarditis; pulmonary tuberculosis.

**Figure 5:**
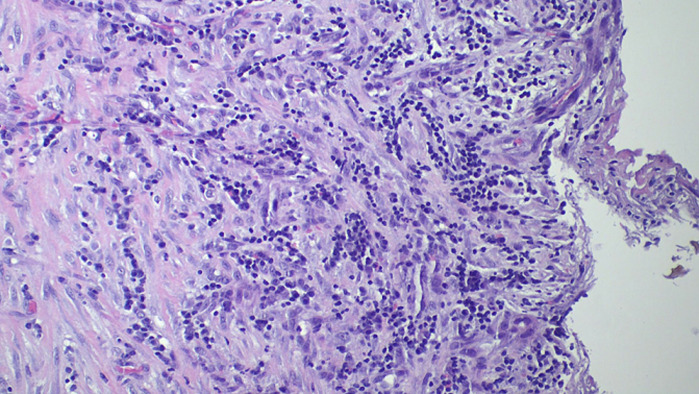
H & E stain slide of the pericardium

**Figure 6:**
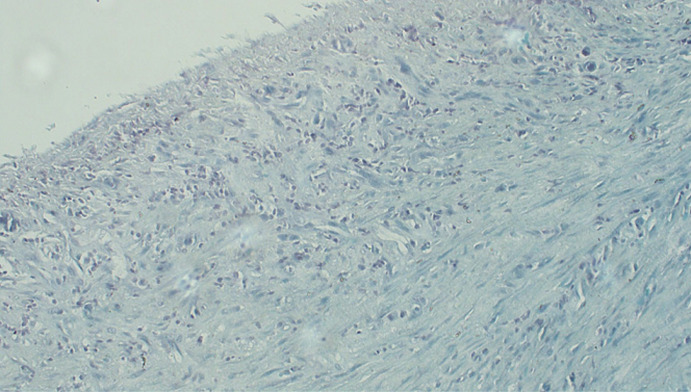
ZN stain histopathology slide of the pericardium

**Therapeutic interventions:** pericardiectomy done, thick fibrotic pericardium encasing all the heart chambers was seen, excision of anterior and left pericardium done, pus and caseous material drained. Left pleural was normal. Post operative diagnosis: TB constrictive pericarditis; bilateral TB empyema thoracis. Histopathology analysis of Pericardial tissue showed: H & E stain showed mixed chronic inflammation and granulation tissue (Figure 5). Impression: non-specific chronic inflammation of the pericardium ZN Stain showed a thick fibrous wall of the pericardium with fibroblast proliferation and mononuclear cells infiltration but no rod-shaped Bacilli for *Mycobacterium tuberculosis* indicating ZN stain is negative (Figure 6).

**Follow up and outcome of interventions:** unfortunately, the patient died about 1-hour post-surgery due to anaesthesia complications.

**Patient´s perspective:** unfortunately, the patient died about 1-hour post-surgery due to anaesthesia complications.

**Informed consent:** written informed consent was obtained.

## Discussion

Bacterial infections are relatively uncommon, but more likely to bring purulent effusions that proceed to cardiac tamponade and pericardial constriction [2]. The most common causes of pericardial infections are viral agents' coxsackievirus, adenovirus, echovirus and retrovirus. The most common organism in bacterial pericarditis is *Staphylococcus aureus* [3,4]. Organisms such as fungal and tuberculosis can also lead to purulent pericarditis. Risk factors include advanced age, diabetes mellitus, untreated infections (pneumonia) extensive burns and immunosuppression [2]. Diagnosing purulent pericarditis to a patient with massive pleural effusion is a challenge unless initial proper physical examination and investigations are done. Some clinical presentations mask pericardial effusion, and this brings delays in immediate and appropriate management to such patients. Purulent pericarditis complication is fatal, especially to immunosuppressive patients despite being infrequent [5]. Immunocompetents respond well to medications when causation is identified. Mortality of purulent pericarditis is high, proper and quick management is recommended [6]. Pertaining to our patient despite a late diagnosis of purulent pericarditis, treatment with empirical antibiotics was started immediately which led to a negative culture from the pericardial and pleural fluid. Normal examination of the oral cavity was inconclusive whether pericarditis was due to the dissemination of bacteria to the mediastinum and pericardium.

After an unresponsive course of antibiotics, anti-tuberculosis drugs: Rifampicin, Isoniazid, Pyrazinamide & Ethambutol (RHZE) were initiated empirically, oral prednisolone was also started as part of the treatment for tuberculous pericarditis. Steroids prevent healing by fibrosis. Few reviews on the use of corticosteroids have shown mortality rate reduction and reaccumulation of fluid after 18-24 months of follow-up, however, the drawback of the reviews is that the small sample size renders the results to be inconclusive [6]. Positive response to RHZE despite negative Acid-Fast Bacillus and Gene Xpert gave us a focus on Tuberculous pericarditis since there was another case report which stated that TB is the most frequent cause of pericardial diseases in the world and in areas where TB is endemic [7]. Pericardiocentesis and pericardial window are treatment modalities of pericardial effusion, as well as prevention of cardiac tamponade [8]. As for our patient, pericardiocentesis was done, and it remained *in situ* for two weeks. Pericardiectomy was chosen as a definitive treatment, which was successively done. Unfortunately, the patient died 5 hours post-surgery with cardiac tamponade as a complication of pericardiectomy. We believe the most likely aetiology of infection in our case was due to TB due to good response from RHZE, it can also be transient bacteremia, a mucosal breach in our patient’s oral cavity with hematogenous spread and seeding of bacteria into the pericardial cavity leading to the condition, although investigations were controversial with a clinical presentation [9].

## Conclusion

Thorough physical examination for patients with massive pleural effusion to rule out pericardial effusion. Serial culture should be performed on such patients if the first result is negative. Empirical treatment with RHZE in resource-limited settings is recommended due to difficulty in identifying the exact cause at a required moment.
